# Correlation of Blood Urea and Creatinine Levels With Thiamin Levels in Type 1 and Type 2 Diabetic Patients

**DOI:** 10.7759/cureus.57022

**Published:** 2024-03-27

**Authors:** Adnan Anwar, Fizza Faisal, Wajeeha Elahi, Ahsan Illahi, Syed Munawar Alam, Syed Tariq Ali Adnan, Syed Asra Batool, Sania Bhagwandas, Atif A Hashmi

**Affiliations:** 1 Physiology, Hamdard College of Medicine and Dentistry, Karachi, PAK; 2 Internal Medicine, Essa General Hospital, Karachi, PAK; 3 Medicine, Ziauddin University, Karachi, PAK; 4 Nephrology, Hamdard University Hospital, Karachi, PAK; 5 Community Medicine, Field Epidemiology Training Program, Sindh Government Hospital, Karachi, PAK; 6 Biochemistry, Fatima Jinnah Dental College, Karachi, PAK; 7 Community Medicine, Karachi Medical and Dental College, Karachi, PAK; 8 Medicine, Hamdard College of Medicine and Dentistry, Karachi, PAK; 9 Medicine, Jinnah Sindh Medical University, Karachi, PAK; 10 Pathology, Liaquat National Hospital and Medical College, Karachi, PAK

**Keywords:** t2dm, t1dm, diabetes type 1, diabetes type 2, creatinine, urea, diabetes mellitus, thiamin

## Abstract

Introduction

Serum urea and creatinine levels are the most commonly recognized parameters for evaluating renal impairment in patients with diabetes mellitus (DM). Therefore, this study evaluated the correlation between urea and creatinine levels and thiamin levels in patients with type 1 DM (T1DM) and type 2 DM (T2DM).

Methods

This multi-center, cross-sectional study was conducted at diabetic outpatient clinics in Karachi. The duration of the study was six months, from 1^st ^January 2023 to 30^th ^June 2023. A total of 60 patients were enrolled and divided into two groups, i.e., T1DM and T2DM, each containing 30 patients of both genders between the ages of 24 and 42 years. Demographic data and biochemical variables, such as urea, creatinine, random blood sugar, fasting blood sugar, hemoglobin A1c, and serum thiamin levels, were assessed. The Mann-Whitney U test and independent t-test were used to associate the means between the two study groups. The chi-square test and Spearman’s correlation coefficient were used to determine the associations between the variables and T1DM and T2DM.

Results

The study results revealed that patients with T2DM had a significantly higher frequency of hypertension (p = 0.039), neuropathy (p = 0.038), and coronary artery disease (p = 0.010) than those with T1DM, in both genders. The level of serum thiamin was found to be significantly higher (p < 0.001) in T2DM (14.8 ± 4.82) than in T1DM patients (7.34 ± 1.90). Similarly, serum creatinine was higher in T2DM than in T1DM patients (0.83 ± 0.12 vs. 0.76 ± 0.17, p = 0.025). Moreover, the correlation of urea and creatinine with thiamin levels in T1DM and T2DM patients revealed that in T1DM and T2DM patients, urea and creatinine showed an insignificant positive correlation with thiamin levels.

Conclusion

We found a significantly higher level of serum creatinine and thiamin levels in T2DM patients than in T1DM; however, there was no significant correlation between urea and creatinine levels and thiamin status in T1DM and T2DM patients. Therefore, we conclude that although serum urea, creatinine, and serum thiamin are important disease biomarkers in diabetic patients, there is no correlation between them.

## Introduction

Diabetes mellitus (DM) prevalence remained to rise from 6.4% in 2010 to 7.7% by 2030, with 285 million people affected globally, representing 6% of the adult population aged 20-79. In 2030, 438 million people worldwide are expected to be diagnosed with DM, with a prevalence of approximately 8%. According to estimates, 438 million individuals will have received a DM diagnosis by 2030, increasing the disease’s overall incidence to approximately 8% [1].

DM is a condition distinguished by an increase in blood sugar levels because of oxidative stress and inflammation, affecting various organs, including the kidneys, retina, and cardiovascular system [2]. DM is classified as type 1 DM (T1DM) and type 2 DM (T2DM). T1DM develops due to the autoimmune disorder of pancreatic beta cells and is caused by the malfunction of beta cells, leading to insulin production failure, whereas T2DM, which is more widespread, is caused by the emergence of insulin resistance and reduced insulin receptor sensitivity [3]. Low levels of thiamin in the body, found in diabetes, affect carbohydrate metabolism. Both T1DM and T2DM patients exhibit amplified renal clearance of thiamin [4].

Thiamin comprises a pyrimidine and thiazole ring connected by a methylene bridge, making it a water-soluble vitamin [5]. Various food sources contain thiamin, a necessary nutrient. However, several factors may contribute to the variance in serum thiamin levels, including high temperatures and pH levels, diuretic usage, high-calorie foods containing carbohydrates, long-term alcohol use, pyrexia, excessive exercise, lactation and pregnancy, stress, and trauma [6,7]. As a necessary coenzyme, thiamin is also essential to the different stages of transitional metabolism. Thiamin influences endothelial function by acting as an antioxidant and exhibiting anti-inflammatory characteristics. Free thiamin monophosphate, thiamin diphosphate, thiamin triphosphate, and adenosine thiamin triphosphate are some of the forms that may be taken in. Thiamine is considered vital to children’s growth and plays a significant role in lipid metabolism [8]. Adult men should consume 1.0-1.4 mg of thiamin daily, whereas adult women should consume 0.8-1.1 mg daily [9].

Biochemical markers are crucial for diagnosing, assessing, and selecting treatments that enhance clinical outcomes. Serum assessment of renal function biomarkers, including creatinine, urea, uric acid, and electrolytes, is regularly employed as an alternative to urine analysis, which can be quite uncomfortable for patients [10]. Blood urea nitrogen (BUN), which quantifies the quantity of urea nitrogen in the blood and is closely associated with kidney excretory function, is a crude and indirect indicator of renal function [10]. Tests for creatinine quantify the blood level of creatinine phosphate and determine problems with renal function. An increase in serum urea and creatinine levels indicates renal failure, although these parameters are good markers of a healthy kidney [11]. The most extensively used and widely recognized metrics for evaluating renal function are serum creatinine and BUN [11,12].

Serum creatinine and urea abnormalities, macroalbuminuria (greater than 300 mg of proteins, primarily albumin) in a 24-hour urine sample, a reduction in glomerular function rate, hypertension, and a significant risk of cardiovascular morbidity and mortality are clinical indicators of diabetic nephropathy [13]. Serum urea and creatinine levels, two biomarkers for diabetic nephropathy, are increased in uncontrolled diabetics with hyperglycemia, which is related to the extent of renal injury. Therefore, these levels can predict the progression of end-stage kidney disease [13,14]. Skeletal muscle releases creatinine, the breakdown product of creatinine phosphate, at a constant rate. The proximal tubule secretes a small amount of it into the glomerular filtrate, and the glomerulus filters it [15,16]. It has remained unrevealed; therefore, people with diabetes have a lower thiamin status than those with healthy metabolic processes for glucose [17]. Therefore, this study assessed and evaluated the correlation between serum urea, creatinine levels, and thiamin status in patients with T1DM and T2DM.

## Materials and methods

Patients and methods

This multi-center, cross-sectional study was conducted on a diabetic outpatient in Karachi using a convenient non-probability sampling technique. The duration of the study consisted of six months, from 1st January 2023 to 30th June 2023. Ethical approval was obtained from Essa General Hospital (approval number: Essa/81/2022). A total of 60 participants were enrolled and divided into two groups, T1DM and T2DM, each containing 30 patients. All subjects with T1DM and T2DM of both sexes between the ages of 24 and 42 years were included in the study. However, diuretic users, those with end-stage renal disease, individuals with major concomitant medical disorders such as chronic liver disease/cirrhosis, gastrointestinal or other malignancies, or those who had undergone transplant surgery were excluded from the study. Moreover, patients undergoing chemoradiation were also excluded from the study. There was no control group involved in the study. After participants provided their written informed consent, demographic data, such as gender, age, and any co-occurring diseases, were collected.

Serum biochemical testing

Heparinized tubes containing blood samples were collected from the multi-center diabetes clinics in Karachi. The drawn blood samples were instantly centrifuged for 20 minutes at 2000 rpm in a non-heparinized tube. The clear supernatant serum was used to assess several biochemical diagnostic variables, such as urea, creatinine, random blood sugar (RBS), fasting blood sugar (FBS), hemoglobin A1c (HbA1c), and serum thiamin levels in the blood. The individual’s right arm’s blood pressure was measured twice: once while seated and once while standing. For every individual, the mean of two measurements taken at intervals of five minutes was noted.

Data analysis

Data were entered and analyzed using SPSS Statistics version 26.0 (IBM Corp. Released 2019. IBM SPSS Statistics for Windows, Version 26.0. Armonk, NY: IBM Corp). Descriptive statistics were expressed as means and standard deviations, whereas gender and comorbidities were documented as frequencies and percentages. The Mann-Whitney U test and independent t-test were used to compare the means between the two study groups. The chi-square test and Spearman correlation coefficient were used to determine the associations between variables and T1DM and T2DM. The statistical significance level was set at <0.05.

## Results

Demographic characteristics of the study population

A total of 60 patients with T1DM and T2DM were studied; among them, 26.7% (n = 8) males and 73.3% (n = 22) females had T1DM, whereas 33.3% (n = 10) males and 66.7% (n = 20) females had T2DM, with no statistically significant difference between them (p = 0.573). There were 36.7% (n = 11) patients with T1DM who had hypertension and 63.3% (n = 19) did not, whereas 63.3% (n = 19) T2DM patients had hypertension and 36.7% (n = 11) did not, while a statistically significant difference was found between them (p = 0.039). Out of 60 diabetic patients, 6.7% (n = 2) T1DM patients had neuropathy and 93.3% (n = 28) did not; however, 26.7% (n = 8) T2DM patients had neuropathy and 73.3% (n = 22) did not, demonstrating the significant difference between them (p = 0.038). Similarly, 6.7% (n = 2) T1DM patients suffered from coronary artery disease and 93.3% (n = 28) did not, whereas 33.3% (n = 10) T2DM patients had coronary artery disease and 66.7% (n = 20) did not, while a significant association was found between them (p = 0.010), as shown in Table 1.

**Table 1:** Demographic details of T1DM and T2DM diabetic patients (n = 60) *p-value significant as <0.05. The data has been presented as n, %. T1DM: type 1 diabetes mellitus, T2DM: type 2 diabetes mellitus

Variables			n	%	p-value
Gender	Male	Type 1	8	26.7	0.573
		Type 2	10	33.3	
	Female	Type 1	22	73.3	
		Type 2	20	66.7	
Hypertension	Yes	Type 1	11	36.7	0.039*
	Yes	Type 2	19	63.3	
	No	Type 1	19	63.3	
	No	Type 2	11	36.7	
Neuropathy	Yes	Type 1	2	6.7	0.038*
	Yes	Type 2	8	26.7	
	No	Type 1	28	93.3	
	No	Type 2	22	73.3	
Coronary artery disease	Yes	Type 1	2	6.7	0.010*
	Yes	Type 2	10	33.3	
	No	Type 1	28	93.3	
	No	Type 2	20	66.7	

The association of demographics and blood pressure among diabetic patients showed that the mean ages of T1DM and T2DM subjects were 24.20 ± 6.39 and 42.73 ± 10.51 years, respectively, representing a significant relationship (p < 0.001). The mean body mass index (BMI) of T1DM and T2DM patients was 15.6 ± 2.87 and 31.85 ± 5.63 kg/m^2^, respectively, and a significant association was found between them (p < 0.001). Likewise, the mean duration of T1DM and T2DM was found to be 4.06 ± 1.94 and 8.93 ± 2.80 years, respectively. Furthermore, the means of systolic blood pressure were observed to be 129.66 ± 12.99 mm Hg in T1DM and 133.66 ± 12.72 mm Hg in T2DM patients; for diastolic blood pressure, it was 89.66 ± 13.51 mm Hg in T1DM and 91.0 ± 10.93 mm Hg in T2DM patients; and 77.3±8.65 beats per min in T1DM and 78.73 ± 5.57 beats per min in T2DM patients for heart rate, respectively, and an insignificant association (p > 0.05) was observed between them. All variables, however, were shown to have higher mean levels in T2DM patients compared to T1DM patients across all age groups, as shown in Table 2.

**Table 2:** The association of demographics and blood pressure in T1DM and T2DM patients *p-value significant as <0.05. The data has been presented as mean and standard deviation. BMI: body mass index, T1DM: type 1 diabetes mellitus, T2DM: type 2 diabetes mellitus

Variables		Mean	Standard deviation	p-value
Age (years)	Type 1	24.20	6.39	<0.001*
	Type 2	42.73	10.51	
BMI (kg/m^2^)	Type 1	15.6	2.87	<0.001*
	Type 2	31.85	5.63	
Duration of diabetes (years)	Type 1	4.06	1.94	<0.001*
	Type 2	8.93	2.80	
Blood pressure, systolic (mm Hg)	Type 1	129.66	12.99	0.233
	Type 2	133.66	12.72	
Blood pressure, diastolic (mm Hg)	Type 1	89.66	13.51	0.676
	Type 2	91.0	10.93	
Heart rate (beats per minute)	Type 1	77.3	8.65	0.449
	Type 2	78.73	5.57	

Differences in serum biochemical markers in T1DM and T2DM groups

As the association of blood glucose levels and thiamin levels among patients with T1DM and T2DM has been observed, the mean HbA1c level was found to be 7.49 ± 0.62% in T1DM and 9.38 ± 1.97% in T2DM, with a significant association between them (p < 0.001). Likewise, the mean FBS was reported to be 151.3 ± 46.03 mg/dl and 211.7 ± 72.1 mg/dl in patients with T1DM and T2DM, respectively; however, a significant association was found between them (p < 0.001). The results further revealed that the mean of RBS was reported as 268.3 ± 36.5 mg/dl and 282.5 ± 45.5 mg/dl in T1DM and T2DM patients, respectively, with an insignificant difference between them (p = 0.189). The level of serum thiamin was found in T1DM patients at 7.34 ± 1.90 ng/mL and 14.8 ± 4.82 ng/mL in T2DM patients, whereas a significant association was observed between them (p < 0.001). In addition, it has been noted that patients with T1DM had lower levels of all variables than those with T2DM. The mean urea level in blood had an insignificant association between T1DM and T2DM (27.23 ± 7.39 vs. 28.4 ± 3.78, p = 0.229). Additionally, the mean creatinine level in blood had a significant association between T1DM and T2DM (0.76 ± 0.17 vs. 0.83 ± 0.12, p = 0.025), as shown in Table 3.

**Table 3:** The association of blood glucose levels, urea, creatinine, and thiamin levels among T1DM and T2DM patients. *p-value significant as <0.05. The data has been presented as mean and standard deviation. RBS: random blood sugar, HbA1c: glycated hemoglobin, FBS: fasting blood sugar, T1DM: type 1 diabetes mellitus, T2DM: type 2 diabetes mellitus

Variables		Mean	Standard deviation	p-value
HbA1c (%)	Type 1	7.49	0.62	<0.001*
	Type 2	9.38	1.97	
FBS (mg/dl)	Type 1	151.3	46.03	<0.001*
	Type 2	211.7	72.1	
RBS (mg/dl)	Type 1	268.3	36.5	0.189
	Type 2	282.5	45.5	
Serum thiamin (ng/ml)	Type 1	7.34	1.90	<0.001*
	Type 2	14.8	4.82	
Blood urea (mg/dl)	Type 1	27.23	7.39	0.229
	Type 2	28.4	3.78	
Serum creatinine (mg/dl)	Type 1	0.76	0.17	0.025*
	Type 2	0.83	0.12	

Correlation of serum urea and creatinine with thiamin levels in T1DM and T2DM groups

The study assessed the correlation of urea and creatinine with thiamin levels in T1DM and T2DM patients and revealed that in the T1DM group, urea (r = 0.205, p = 0.276) and creatinine (r = 0.288, r = 0.123) showed an insignificant positive correlation with thiamine levels. In the T2DM group, urea (r = 0.094, p = 0.622) and creatinine (r = 0.120, p = 0.529) levels also showed an insignificant positive correlation with thiamin levels (Table 4).

**Table 4:** The correlation of urea and creatinine levels with thiamin status in T1DM and T2DM patients r: Pearson correlation coefficient, T1DM: type 1 diabetes mellitus, T2DM: type 2 diabetes mellitus

Groups	Variables	Thiamin	
		r	p-value
T1DM (n=32)	Urea	0.205	0.276
	Creatinine	0.288	0.123
T2DM (n=32)	Urea	0.094	0.622
	Creatinine	0.120	0.529

## Discussion

Blood markers such as creatinine and urea are useful for evaluating renal function. Elevated urea and creatinine levels in the blood may indicate impaired renal function. Therefore, this study demonstrated a correlation between urea and creatinine levels and thiamin status in patients with T1DM and T2DM.

A prospective study evaluated blood creatinine and thiamin levels in individuals with diabetes. In both types of diabetes, a strong and positive correlation (p < 0.001) was observed between urine thiamin and serum creatinine levels [4]. The present study found that thiamin levels were not substantially correlated with urea and creatinine levels in either type of diabetes (p > 0.05), contradicting the findings of the previous study.

Similarly, findings from another study indicated statistically significant differences in the means of all basic characteristics, excluding height and temperature, among the three study groups. These baseline characteristics encompassed age, BMI, weight, heart rate (p < 0.001 for all), systolic blood pressure (p = 0.001), and diastolic blood pressure (p = 0.002). Specifically, patients with T1DM exhibited a younger age and a lower BMI and weight than those with T2DM or the control group. In contrast, patients with T1DM and T2DM manifested higher systolic and diastolic blood pressure as well as a higher heart rate when compared with the control group [18]. Our study was partially consistent with the above-reported study and indicated that patients with T1DM were younger than those with T2DM, with a significant difference among them (p < 0.001). However, insignificant associations were observed in the means of systolic blood pressure (p = 0.233), diastolic blood pressure (p = 0.676), and heart rate (p = 0.449).

Likewise, a comparative analysis of hematological and biochemical markers in diabetic patients was performed at many diabetic outpatient centers in Karachi. The results of the study showed that patients with T2DM had significantly higher mean levels of HbA1c, RBS, and FBS than either T1DM patients or the control group (p < 0.001 for all). In addition, the results showed that serum thiamin levels were significantly lower in patients with T1DM or T2DM than in controls (14.89 ± 4.82 and 7.35 ± 1.90 vs. 69.56 ± 12.75, p < 0.001) [19]. According to another study conducted in 2015, there was a significant increase in glucose levels (p = 0.001) between patients with T1DM and controls [19]. The study findings also demonstrated that patients with T1DM and T2DM had significantly higher HbA1c values than controls [19]. In addition, a 2003 study discovered that patients without diabetes had lower HbA1c levels than those with diabetes (p = 0.002). Furthermore, HbA1c is a very helpful and targeted diabetic screening and diagnostic tool [20]. In this study, the mean FBS and HbA1c levels were substantially higher in patients with T2DM than in those with T1DM (p < 0.001). With regard to thiamin levels, noticeably greater amounts were found in T2DM patients than in T1DM patients (14.8 ± 4.82 vs. 7.34 ± 1.90, p < 0.001).

Furthermore, a different study found statistically significant differences in the means of blood glucose levels and all lipid profiles, including serum thiamin triglycerides, FBS, HbA1c, and RBS, between controls, T1DM, and T2DM (p < 0.001) [21]. These findings were partially consistent with the present study and showed that statistically significant differences were noticed among T1DM and T2DM in the means of HbA1c, FBS, and serum thiamin levels (p < 0.001); however, a statistically insignificant association was observed in the means of RBS (p = 0.189).

Urea and creatinine serum levels serve as valuable prognostic indicators and predictors of renal impairment in individuals with diabetes [22]. Similarly, a study assessed the serum urea and creatinine levels of diabetic and non-diabetic patients in a tertiary hospital. The results of the study showed that the mean blood sugar levels in control were 88.05 ± 8.96 and 124.67 ± 8.94 for fasting and postprandial, respectively, whereas in the diabetes group, the values were 133.88 ± 68.993 and 168.01 ± 74.87, respectively. As a result, compared with the non-diabetic control participants, the mean fasting and postprandial blood sugar levels were higher in the diabetes subjects. In the control group, the mean urea level was 18.31 ± 4.55, but in the diabetes group, it was 29.22 ± 20.32. The mean creatinine levels in diabetics were 1.13 ± 0.77 and in controls were 0.89 ± 0.21. Thus, the diabetics’ mean serum urea and creatinine levels were noticeably greater than those of the non-diabetic control group. Serum urea and blood sugar levels, both fasting and postprandial, showed a significant positive correlation of 0.76 and 0.83, respectively. However, a slight positive correlation was found between blood sugar and serum creatinine levels, both during fasting and after meals (i.e., 0.28 and 0.40, respectively) [23]. Consequently, inadequately managed blood glucose levels increase serum urea and creatinine levels, increasing the risk of developing diabetic nephropathy. This supports the results of other studies that found that hyperglycemia is one of the main contributors to increasing renal impairment [24]. When there is a renal injury, the urea level increases. When a diabetic patient has high blood sugar and an increase in blood urea level, this suggests renal impairment. According to research by Anjaneyulu et al. [24], increases in blood creatinine and urea levels in diabetic rats suggest increased renal impairment [24]. In this study, by assessing both types of patients with diabetes, a significant association was observed in the mean FBS (151.3 ± 646.03 and 211.7 ± 72.1, p < 0.001). The mean urea level in T1DM was found to be 27.23 ± 7.39, whereas in T2DM it was found to be 28.4 ± 3.78. The mean creatinine levels in T1DM were found to be 0.76 ± 0.17, and in T2DM, they were found to be 0.83 ± 0.12. Therefore, it was evident that the urea and creatinine levels in T2DM were apparently higher than those in T1DM. Moreover, a weak positive correlation was observed between urea, creatinine, and thiamin levels in patients with T1DM and T2DM.

Limitations

The study has a few limitations. First, although it was a multi-center study, the sample size was limited. Moreover, we did not seek association with other biochemical markers with serum thiamin. In addition, it is important to acknowledge the potential for selection bias in this study, which can be attributed to the use of a non-probability sampling technique and observer bias. Therefore, it is advisable to conduct prospective studies employing a probability sampling method to further investigate this correlation in larger samples, aiming for more accurate and reliable results.

## Conclusions

This study concluded that there was no significant correlation between urea and creatinine levels and thiamin status in T1DM and T2DM patients. Additionally, we found a higher level of serum thiamin and creatinine in T2DM patients than in those with T1DM. Therefore, although serum urea, creatinine, and thiamin are important biochemical markers to monitor in diabetic patients, there seems to be no correlation between them.
